# Unequal recovery of TB notifications after COVID-19 in Colombia, 2018–2022

**DOI:** 10.5588/ijtldopen.26.0142

**Published:** 2026-09-15

**Authors:** S. Valencia-Aguirre, C.A. Castaneda-Orjuela, L.H. Gaitán, C.F. Escobar, B.S. Cervera, A.L. García-Basteiro

**Affiliations:** 1 Barcelona Institute for Global Health (ISGlobal), Hospital Clínic of Barcelona, Universitat de Barcelona, Barcelona, Spain;; 2 Research Group in Epidemiology, University of Antioquia, Medellín, Colombia;; 3 Epidemiology and Public Health Evaluation Group, Universidad Nacional de Colombia, Bogotá, Colombia;; 4 Bogota District Health Secretariat (Secretaria Distrital de Salud de Bogota), Bogota, Colombia;; 5 Parasites and Microbes, Wellcome Sanger Institute, Cambridge, UK;; 6 Centro de Investigação em Saúde de Manhiça (CISM), Manhiça, Mozambique;; 7 Centro de Investigación Biomédica en Red de Enfermedades Infecciosas CIBERINFEC, Barcelona, Spain;; 8 Department of Preventive Medicine and Epidemiology, Hospital Clínic, Barcelona, Spain.

**Keywords:** tuberculosis, case notification, health inequities, public health surveillance, interrupted time series, Latin America

## Abstract

**BACKGROUND:**

The COVID-19 pandemic disrupted TB services and case notifications worldwide, but disaggregated analyses are needed to identify inequities in post-pandemic recovery.

**METHODS:**

We analysed national TB surveillance data from Colombia (January 2018–November 2022) using interrupted time-series models. Monthly notification rates were estimated overall and by sex, age, health insurance scheme, area of residence, and indigenous status. Models quantified the immediate change in March 2020, post-interruption trend changes, and divergence from counterfactual rates projected from pre-pandemic trends.

**RESULTS:**

National notification rates fell by 17.1 cases per million in March 2020, a 45.0% reduction relative to expected values. Notifications subsequently increased and by November 2022 exceeded counterfactual projections by 13.2%. Disaggregated analyses showed heterogeneous recovery. Rates were 19.3% above expected among men versus 3.0% among women, and 13.1% among adults aged ≥15 years versus 4.0% among children aged <15 years. Notifications in the subsidised insurance scheme were ∼26% above expected, whereas those in the contributory scheme remained below expected. Urban rates exceeded projections, while rates for indigenous men remained below.

**CONCLUSION:**

National recovery masked persistent subgroup inequities, highlighting the need for disaggregated surveillance to guide equitable TB control after major health system shocks.

Global crises (including infectious disease outbreaks, economic instability, and environmental stressors) challenge health systems’ capacity to maintain essential services.^1,2^ TB is the leading infectious cause of death worldwide^3^ and is particularly sensitive to such shocks.^4,5^ Prior to the onset of the COVID-19 pandemic, global efforts had achieved substantial progress, with TB incidence declining by 9% and mortality by 14% worldwide between 2015 and 2019.^6^ However, the declaration of COVID-19 in March 2020 triggered a global health and economic crisis that disrupted health systems and reversed gains in TB prevention and control by redirecting resources toward response and constraining essential programmes, including TB services.^7,8^ Global estimates indicate that 1.4 million fewer people received TB treatment in 2020 than in 2019.^9,10^ In the Americas, notifications decreased by 16.8% between 2019 and 2020, interrupting the upward trend observed since 2015 and reverting to levels comparable to 2012.^11^

Colombia showed similar trends: in 2020, 12,582 cases were notified, a 19.2% reduction compared to 2019, alongside a 58% relative increase in advanced disease at diagnosis defined as cases presenting severe clinical or bacteriological indicators consistent with delayed detection.^12^ At the onset of the pandemic, Colombia implemented stringent lockdowns, border closures, and mobility restrictions, followed by phased reopening from September 2020.^13^ In this context, in March 2020 a public health emergency was declared, and in April, the Ministry of Health updated TB guidance promoting broader molecular diagnostics and community-based or virtual directly observed treatment.^14^ Given Colombia´s regional diversity and socio-economic inequalities, the implementation and effects of national TB protocols were unlikely to be uniform across territories.^15,16^ Disaggregated analyses are therefore necessary to identify population groups in which declines in TB notifications persisted or recovery diverged from national patterns, potentially reflecting sustained barriers to timely diagnosis and care. Although declines in TB notifications during COVID-19 have been widely reported, fewer studies have examined longer-term post-interruption trajectories relative to pre-existing trends. In Colombia, TB notifications had shown a sustained upward trajectory prior to 2020, making it essential to determine whether subsequent levels represented true recovery, compensatory rebound due to delayed detection, or a persistent deficit relative to expected patterns.^5,17^ TB and COVID-19 share structural determinants (including poverty, overcrowding, informal labour, and barriers to health care access), highlighting the need for differentiated recovery strategies.^18,19^ These mechanisms are unlikely to operate uniformly across socially and territorially diverse populations, particularly in settings characterised by health system segmentation and marked inequalities.

This study aimed to quantify the immediate disruption and subsequent recovery of TB case notifications following the onset of COVID-19 pandemic in Colombia (2018–2022), and to assess divergence from pre-pandemic trends across population groups defined by sex, age, health insurance regime, area of residence, and indigenous status.

## METHODS

We conducted a retrospective interrupted time-series (ITS) analysis using national TB surveillance data from Colombia between January 2018 and November 2022. The study included all TB cases notified through the national surveillance system during this period.

### Data sources and population

TB notification data were obtained from the Public Health Surveillance System (SIVIGILA).^20^ Demographic variables included sex, age, area of residence, Indigenous status, and health insurance affiliation. Health insurance affiliation was used as a proxy for socio-economic position and differential access to health care.^21^ Colombia’s health system includes two main insurance regimes: contributory, for formally employed or higher-income individuals, and subsidised, state-funded for those without the ability to pay. Population denominators were derived from the official population projections of the National Administrative Department of Statistics (DANE).^22^ COVID-19 case data were extracted from the open-access official reports of the Ministry of Health and Social Protection.^23^ National COVID-19 control measures included an initial period of strict lockdown and mobility restrictions beginning in March 2020, followed by phased reopening.

### Statistical analysis

Monthly age-adjusted TB notification rates were calculated for the overall population and for the following subgroups: sex (women/men), age (<15 years and ≥15 years), area of residence (urban/rural), health insurance regime stratified by sex (contributory/subsidised), and Indigenous population stratified by sex. Rates were expressed as monthly cases per million population. Data for area of residence were excluded for 2018 because SIVIGILA records for that year showed an unexpectedly low number of TB cases with valid area-of-residence classification, close to zero or minimal values, followed in 2019 by a marked increase. This abrupt pattern was considered inconsistent with expected epidemiological variation and more likely to reflect a data-quality or coding problem in the area-of-residence variable for 2018. Therefore, to avoid misclassification bias and distortion of urban-rural trends, 2018 was excluded only by this subgroup. Segmented linear regression models were applied to monthly TB notification rates using an ITS framework to estimate changes before and after the onset of the COVID-19 pandemic in March 2020. For each population group, models estimated 1) the baseline level (intercept) and slope of TB notification rates during the pre-pandemic period; 2) the immediate level change in March 2020; and 3) the slope change, capturing differences in post-pandemic trends relative to pre-pandemic trends. Two-tailed *P* values < 0.05 and 95% confidence intervals were considered statistically significant. Descriptive metrics included mean rates and annualised changes for the pre-pandemic and pandemic/recovery periods. Absolute and relative changes in March 2020 were estimated against expected values under the pre-pandemic trend. Counterfactual projections were extrapolated through November 2022. Observed notification rates in November 2022 were compared with these projections to calculate absolute and relative differences. Model specification was evaluated through sensitivity analyses comparing two alternative change-point definitions: 1) a two-phase model with a single interruption in March 2020 and 2) a three-phase model distinguishing pre-pandemic, lockdown, and recovery periods. Model fit was assessed using likelihood-ratio tests. Analyses were conducted using R software (version 4.4.0).

### Ethical statement

This study used fully anonymised secondary data from national public health surveillance records. No individual identifiers were accessible to the research team. According to national regulations, studies based exclusively on anonymised surveillance data are exempt from formal ethical review. This exemption was confirmed by the Institutional Research Ethics Committee of the National Institute of Health (CEIN) in 2022.

## RESULTS

**Figure 1.**
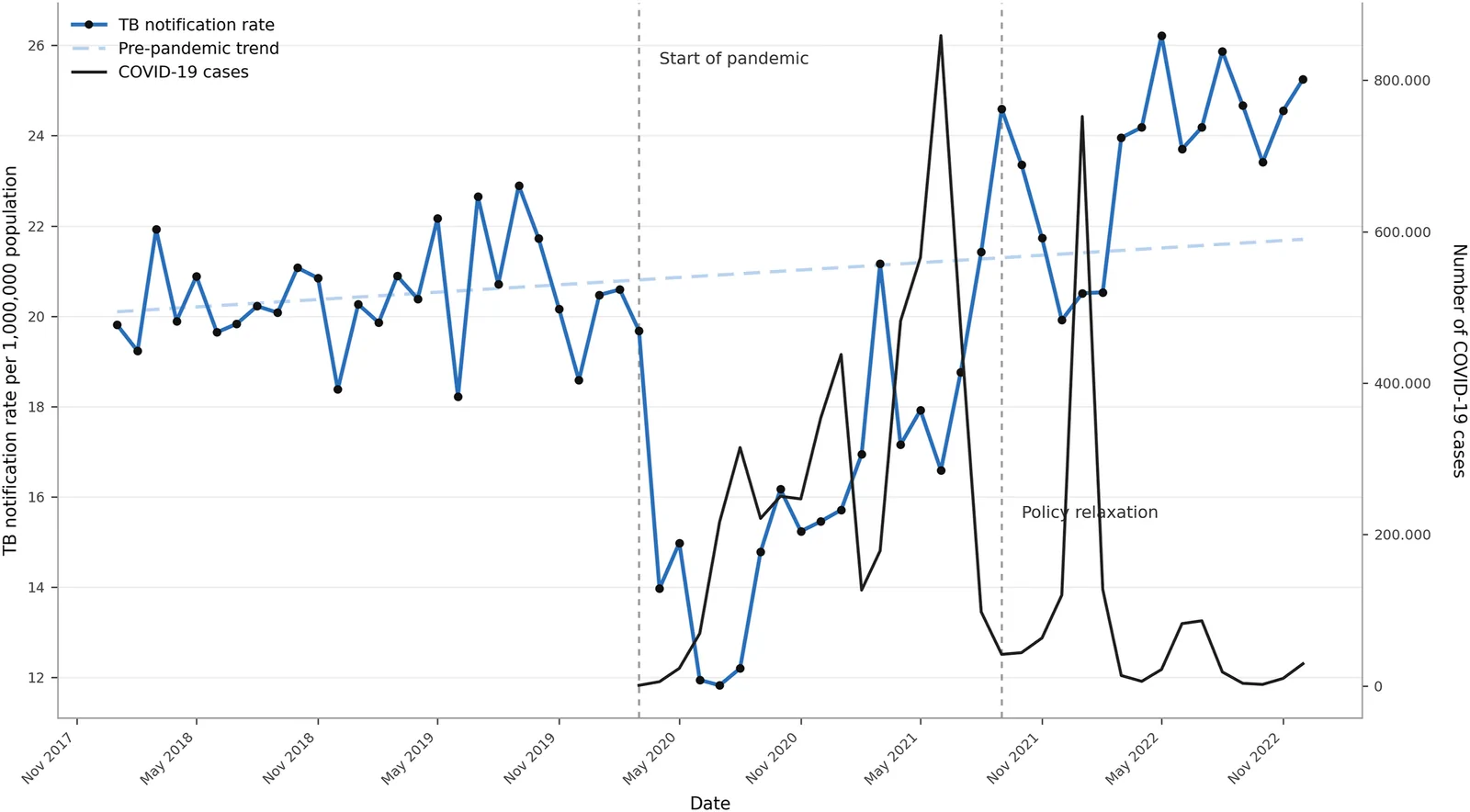
National TB notification rates and COVID-19 cases in Colombia, 2018–2022. Monthly TB notification rates (cases per 1,000,000 population; left axis) and reported COVID-19 cases (right axis) in Colombia from January 2018 to November 2022. The vertical dashed line marks March 2020, defined as the interruption point in the interrupted time-series analysis. A second dashed line indicates the policy relaxation phase. The blue dashed line shows the counterfactual projection based on pre-pandemic trends.

**Figure 2.**
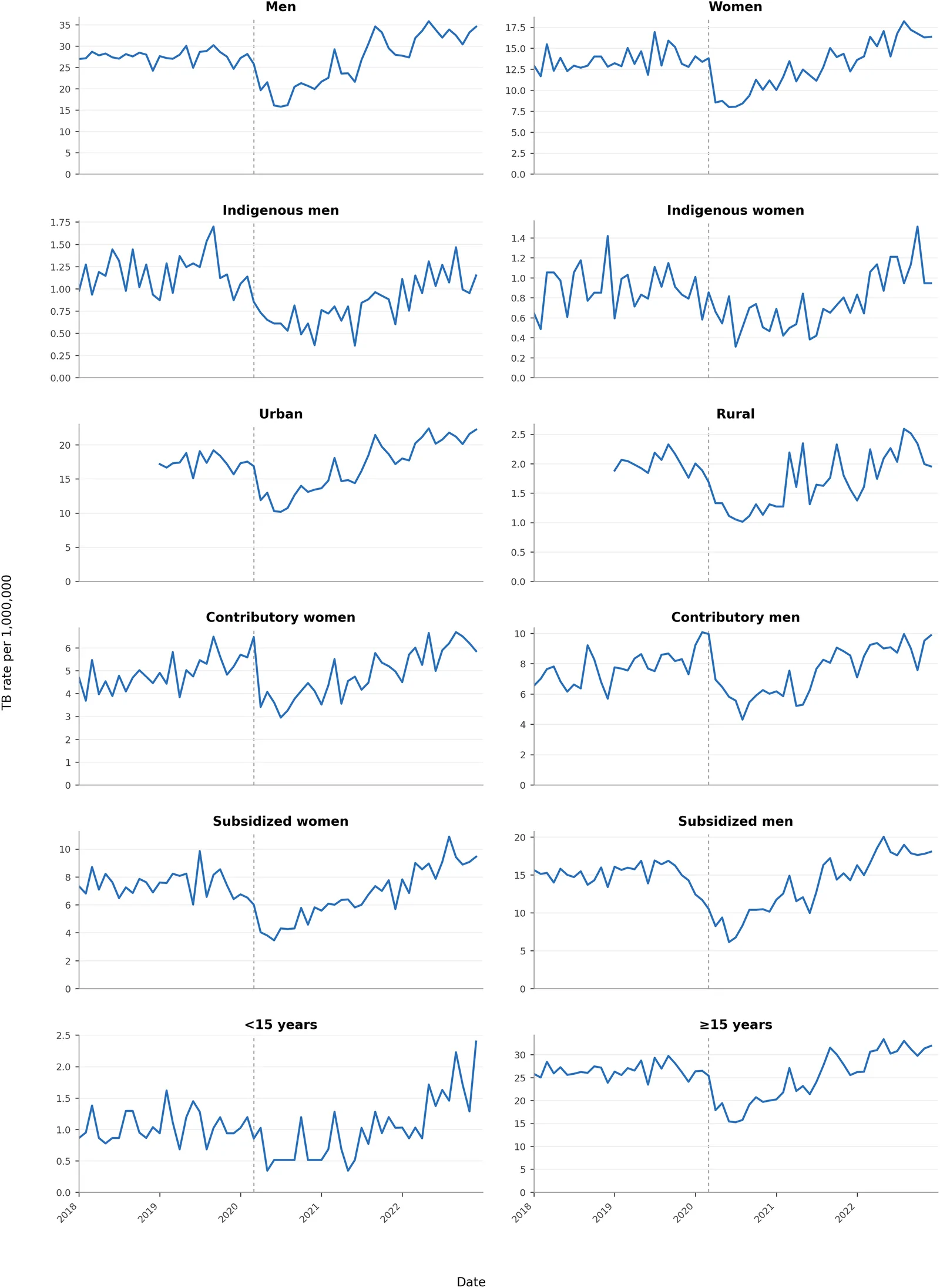
Monthly TB notification rates by population subgroup, Colombia, 2018–2022. Descriptive monthly TB notification rates (cases per 1,000,000 population) stratified by sex (men, women), Indigenous status by sex, area of residence (urban, rural), insurance regime by sex (contributory, subsidised), and age group (<15 years; ≥15 years). The vertical dashed line indicates March 2020, corresponding to the onset of COVID-19–related service disruption.

**Figure 3.**
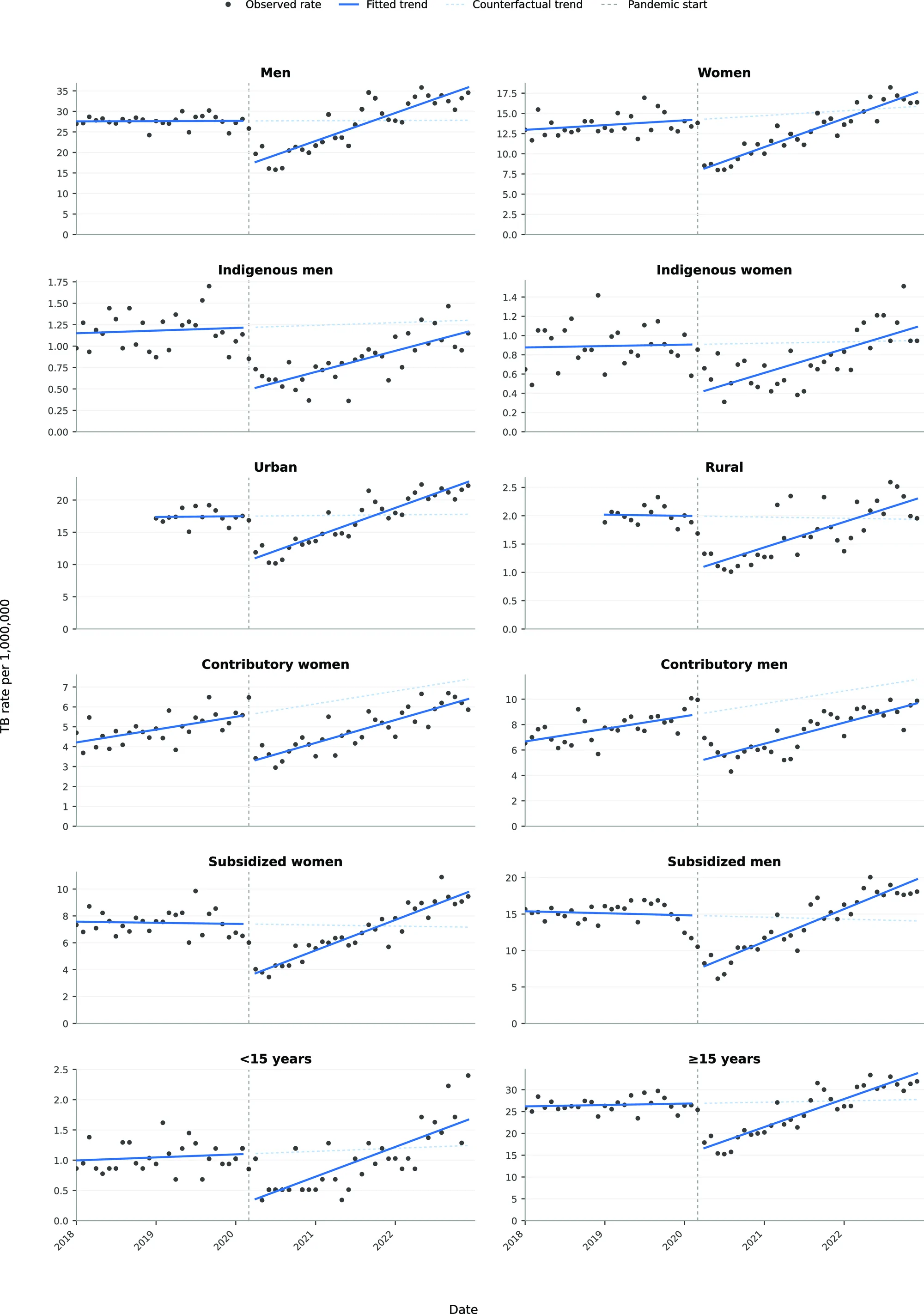
Interrupted time-series (ITS) models of TB notification rates by subgroup, Colombia, 2018–2022. Observed monthly TB notification rates (points) and fitted segmented regression lines for pre-pandemic and pandemic/recovery periods (solid lines) are shown for each subgroup. The dotted line represents the counterfactual trajectory projected from the pre-pandemic trend. The vertical dashed line indicates the interruption point in March 2020.

We assessed model specifications and estimated immediate and post-interruption changes in TB notification trends across subgroups (Figures 1–3; Tables 1 and 2).

**Table 1.** Segmented regression estimates of level, slope, and rate changes in TB notifications before and after the onset of the COVID-19 pandemic, Colombia, 2018–2022.

	Pre-pandemic (January 2018–March 2020)				Pandemic/recovery (April 2020–November 2022)				Interruption effect (March 2020)			
Group	Intercept	Slope	Mean	Annual change	Intercept	Slope	Mean	Annual change	Immediate change (95% CI)	Slope change (95% CI)	Drop in March (n)	Drop in March (%)
Overall	20.1	0.0009	20.4	0.3	1.3	0.0141	19.7	5.1	−17.1 (−20.1, −14.0)***	0.012 (0.008, 0.016)***	9.1	−45.0
Men	27.6	0.0001	27.6	0.1	2.3	0.0187	26.8	6.8	−23.1 (−27.6, −18.7)***	0.017 (0.012, 0.023)***	11.3	−41.0
Women	12.9	0.0016	13.6	0.6	0.2	0.0097	12.9	3.5	−11.3 (−13.6, −9.0)***	0.007 (0.004, 0.010)***	7.2	−43.0
<15 years	1.0	0.0001	1.0	0.1	−0.8	0.0013	1.0	0.5	−1.6 (−2.1, −1.1)***	0.001 (0.000, 0.002)***	0.5	−49.0
≥15 years	26.2	0.0009	26.5	0.3	2.2	0.0176	25.2	6.4	−21.7 (−25.6, −17.8)***	0.015 (0.010, 0.020)***	9.6	−42.0
Subsidised – men	15.4	−0.0007	15.1	−0.3	−2.2	0.0123	13.8	4.5	−16.9 (−19.4, −14.3)***	0.012 (0.009, 0.016)***	8.4	−55.0
Subsidised – women	7.6	−0.0002	7.5	−0.1	−1.4	0.0062	6.8	2.3	−8.3 (−9.7, −6.9)***	0.006 (0.004, 0.008)***	4.0	−53.0
Contributive – men	6.7	0.0027	7.7	1.0	1.5	0.0045	7.5	1.7	−4.0 (−5.8, −2.2)***	0.001 (−0.001, 0.003)	2.8	−36.0
Contributive – women	4.2	0.0018	4.8	0.7	0.7	0.0032	4.9	1.2	−2.7 (−3.9, −1.5)***	0.001 (−0.001, 0.002)	1.5	−35.0
Indigenous – men	1.1	0.0001	1.9	0.0	−0.0	0.0007	0.8	0.3	−1.1 (−1.4, −0.8)***	0.001 (0.000, 0.001)*	0.3	−28.0
Indigenous – women	0.9	0.0000	0.9	0.0	−0.1	0.0007	0.8	0.3	−0.9 (−1.3, −0.5)***	0.001 (0.000, 0.001)*	0.3	−30.0
Urban	17.4	0.0003	17.4	0.1	5.5	0.0121	16.9	4.4	−10.8 (−13.3, −8.2)***	0.011 (0.003, 0.018)**	7.2	−41.0
Rural	2.0	−0.0001	2.0	−0.0	0.5	0.0012	1.7	0.5	−1.4 (−1.8, −0.9)***	–	–	–

All rates are expressed as cases per 1,000,000 population per month. The pre-pandemic period covers January 2018–March 2020. The pandemic/recovery period covers April 2020–November 2022. March 2020 was defined as the interruption point in the interrupted time-series (ITS) analysis. Intercept represents the estimated baseline notification rate at the start of each period. Slope represents the estimated monthly change in notification rate (cases per 1,000,000 population per month). Mean indicates the average monthly notification rate within each period. Annual change corresponds to the slope multiplied by 12. Immediate change (March 2020) represents the estimated level change at the interruption point derived from the segmented regression model, with 95% confidence intervals (CIs). Slope change represents the difference between post-pandemic and pre-pandemic slopes. Positive values indicate acceleration in notification rates after March 2020, whereas negative values indicate continued decline. Drop in March (n) and drop in March (%) reflect the estimated reduction in notification rates at the interruption point relative to the counterfactual projection based on pre-pandemic trends.

Statistical significance is indicated as follows: *P < 0.05; **P < 0.01; ***P < 0.001.

**Table 2.** Observed and counterfactual TB notification rates in November 2022, Colombia.

Group	Observed rate (November 2022)	Counterfactual rate (November 2022)	Absolute difference	Relative difference (%)
Overall	24.6	21.7	2.9	13.2
Men	33.2	27.8	5.4	19.3
Women	16.3	15.8	0.5	3.0
<15 years	1.3	1.2	0.1	4.0
≥15 years	31.3	27.7	3.6	13.1
Subsidised – men	17.8	14.1	3.7	26.4
Subsidised – women	9.1	7.2	1.9	26.3
Contributory – men	9.5	11.5	−2.0	−17.1
Contributory – women	6.2	7.3	−1.1	−15.4
Indigenous – men	1.0	1.3	−0.4	−26.9
Indigenous – women	1.0	1.0	0.0	0.0
Urban	21.6	17.8	3.8	21.4
Rural	2.0	1.9	0.1	2.6

Observed rate corresponds to the reported TB notification rate per 1,000,000 population in November 2022. Counterfactual rate represents the expected value projected from the pre-pandemic trend under a no-COVID scenario. Absolute difference is calculated as observed minus counterfactual rate. Relative difference (%) expresses the proportional deviation from the counterfactual estimate. All rates are expressed per 1,000,000 population.

### Sensitivity analysis

The likelihood-ratio test did not indicate a statistically significant improvement in model fit when including a third period (residual sum of squares: 235.4 vs. 180.9). Although the three-phase model reduced unexplained variance, the improvement was not statistically significant and did not justify the additional change point. Accordingly, the two-period model was retained, defining a pre-pandemic phase (January 2018–March 2020) and a pandemic/recovery phase (April 2020–November 2022).

### Interrupted time series

National and subgroup trends, segmented regression estimates, counterfactual comparisons, and ITS models are shown in Figures 1–3 and Tables 1–2. At the national level, prior to March 2020, TB notification rates averaged 20.4 cases per million population and showed a modest upward trajectory (annual change: 0.3 cases per million). In March 2020, a significant immediate reduction of −17.1 cases per million was observed (95% CI −20.1 to −14.0; *P* < 0.001), corresponding to a 45.0% decline relative to expected values based on pre-pandemic trends. Following this interruption, notification rates increased with a steeper slope (post-pandemic annual change: 5.2 cases per million). By November 2022, observed rates exceeded counterfactual projections by 2.9 cases per million (13.2%) (Table 2). Sex-stratified analyses showed that before the pandemic, notification rates among men were approximately twice those among women (27.6 vs. 13.6 cases per million), with limited pre-pandemic annual change in both groups (Figure 2; Table 1). In March 2020, both sexes experienced immediate reductions (−23.1 among men and −11.3 among women; *P* < 0.001), corresponding to proportional declines of 41.0% and 43.0%, respectively. Post-pandemic slope changes were larger among men (0.017 vs. 0.007 cases per million per month; *P* < 0.001 for both), resulting in greater divergence from counterfactual projections by November 2022 (19.3% above expected levels in men vs. 3.0% in women) (Table 2). Age-specific analyses revealed distinct dynamics (Figure 2; Table 1). Among children (<15 years), baseline notification rates were low (mean: 1.05 cases per million), and the immediate reduction in March 2020 was proportionally the largest observed (−49.0%). Subsequent increases were modest, and by November 2022 observed rates were only slightly above counterfactual projections (4.0%) (Table 2). In contrast, among individuals aged ≥15, baseline rates were higher (26.5 cases per million), and the immediate decline (−42.0%) was followed by a sustained upward trend (annual change: 6.4 cases per million), resulting in observed rates 13.1% above projected levels. Patterns also differed by insurance regime, Indigenous status, and area of residence (Figures 2–3; Tables 1 and 2). In the subsidised regime, both men and women experienced immediate declines exceeding 50.0%, followed by significant post-pandemic increases. By November 2022, observed rates were approximately 26.0% above counterfactual projections in both sexes (Table 2). In contrast, in the contributory regime, although immediate reductions were significant (−36.0% among men; −35.0% among women), post-pandemic slope changes were not significant, and rates remained below expected levels by November 2022 (−17.1% among men; −15.4% among women). Differences between regimes were therefore driven primarily by recovery trajectories rather than the magnitude of the initial decline.

Among Indigenous populations, immediate reductions ranged from 28% to 30.0%, followed by modest post-pandemic slope increases (Table 1). By November 2022, notification rates among Indigenous men remained 26.9% below counterfactual projections, whereas Indigenous women approximated expected trends (Table 2). Differences were also observed by area of residence. Urban areas experienced significant post-pandemic acceleration and exceeded counterfactual projections by 21.4%, whereas rural areas showed no significant slope change and remained close to projected levels (2.6% above counterfactual). Across subgroups, heterogeneity in post-pandemic patterns was primarily attributable to differences in slope changes rather than variation in the magnitude of the initial interruption.

## DISCUSSION

This study applied an ITS analysis (2018–2022) to quantify the impact of the COVID-19 pandemic on TB notifications in Colombia. Nationally, notifications decline sharply in March 2020 (−17.1 cases per million; *P* < 0.001), 45% below expected values, consistent with global trends.^5,24–26^ This decrease likely reflects service disruptions, reallocation of diagnostic resources to COVID-19, and reduced health care-seeking during lockdowns.^14^ Given the natural history of TB, the magnitude of the decline suggests under-detection rather than a true reduction in incidence. Following March 2020, notification rates exhibited a significant positive slope change (*P* < 0.001), indicating recovery. By November 2022, national rates exceeded counterfactual projections by 13.2%, a rebound comparable to patterns reported in Brazil and Zambia after WHO’s global ‘catch-up’ strategy in 2021–2022.^25,26^ In Colombia, this recovery coincided with implementation of the National TB Recovery Plan 2020, expansion of molecular diagnostic capacity, and intensified active case-finding.^12,14^ This post-pandemic increase should be interpreted cautiously. Higher-than-expected notifications after a major service disruption do not necessarily indicate a true increase in TB incidence or complete programme recovery. They may reflect restored diagnostic capacity, catch-up detection of missed or delayed cases, and possible transmission effects from delayed diagnosis. Surveillance data cannot disentangle these processes, but the 2020 decline and subsequent rebound suggest that part of the increase reflects recovery of case detection and delayed ascertainment.

The pandemic’s impact and recovery were not uniform across population groups.^27^ By November 2022, notification rates among men exceeded counterfactual projections by 19.3%, compared with 3.0% among women. These differences mainly reflect steeper post-pandemic slope among men. Although the mechanisms underlying these sex patterns cannot be directly assessed here, these findings are consistent with literature suggesting that gender-related barriers may shape access to TB diagnosis and care during health system disruptions. Global modelling suggests that pandemic-related disruptions may have disproportionately affected women through intensified caregiving responsibilities, reduced autonomy, and financial barriers. Evidence from Eastern Europe and Central Asia similarly indicates gender-specific constraints in TB diagnosis and care.^17^ In Colombia, structural gender inequities may have contributed to slower recovery in TB detection among women, but this interpretation should be considered hypothesis-generating. Age patterns revealed further heterogeneity. Children under 15 years experienced the largest proportional decline (−49.0%) despite having low baseline rates (1.1 per million), suggesting near-complete interruption of paediatric case detection at the onset of the pandemic. Recovery remained limited, with rates only 4.0% above counterfactual projections by late 2022. In contrast, adults (≥15 years) experienced a 42% decline followed by a sustained upward trajectory, reaching 13.1% above expected levels by November 2022. These findings align with global evidence indicating that paediatric TB services were particularly vulnerable to pandemic-related disruption, possibly due to interruptions in contact tracing, reduced clinical suspicion, or barriers to accessing routine health services.^28^

Socio-economic stratification, approximated by health insurance regime, also shaped recovery patterns. The subsidised regime, which covers individuals without formal employment, exhibited sharper initial declines (exceeding 50.0%) but also stronger recovery, with rates approximately 26.0% above counterfactual projections by 2022. Conversely, the contributory regime showed smaller initial reductions but no significant post-pandemic acceleration, and rates remained below expected levels (−17.0% in men; −15.0% in women). The divergence between regimes was therefore driven primarily by differences in recovery trajectories rather than by the magnitude of the initial shock. These patterns are consistent with prior evidence that health system segmentation and socio-economic vulnerability influence TB notification dynamics in Colombia.^10,21,29^ Territorial and ethnic disparities persisted. Urban areas demonstrated significant post-pandemic acceleration and exceeded projected levels by 21.0%, whereas rural areas showed weaker recovery and remained close to expected trends. Among Indigenous populations, notification rates remained below national averages throughout the study period; Indigenous men were 27.0% below projected levels by November 2022, while Indigenous women approximated expected trends. These findings may reflect persistent structural barriers to diagnosis, including geographic isolation, limited laboratory infrastructure, linguistic challenges, and health system fragmentation.^30–35^ Territorial differences in notification rates should therefore be interpreted not only as differences in transmission risk but also as potential indicators of unequal health system capacity.^11^ These findings have practical implications for TB programme governance. National recovery plans should define common goals, minimum standards, and core interventions. However, they should also allow territories to adapt implementation according to epidemiological profiles, health system capacity, and population-specific gaps. A uniform strategy may overlook groups with slower or incomplete recovery. TB programmes should therefore combine national guidance with territorial decision-making.

A key strength is the disaggregated analysis, which identified heterogeneous recovery trajectories across sex, age, socio-economic position, ethnicity, and territory. However, several limitations should be considered. Use of national surveillance data may introduce underreporting bias and limit assessment of subnational heterogeneity. The ITS design estimates changes in notification trends but does not allow direct causal inference about mechanisms underlying subgroup differences. It also assumes linear pre-pandemic trends, which may not fully capture nonlinear dynamics. In addition, the analysis focused on case notifications and did not assess outcomes such as TB mortality, treatment success, or drug resistance. Future research should use multivariable or mixed-effects ITS models to better assess socio-economic and territorial drivers of TB recovery.

## CONCLUSION

The COVID-19 pandemic caused a sharp but temporary decline in TB notifications in Colombia, followed by a heterogeneous recovery. While national rates eventually exceeded counterfactual projections (13.2% above expected levels by November 2022), recovery patterns differed substantially across population groups. Persistent gaps among Indigenous populations, rural residents, women, and individuals in the contributory health system underscore the role of structural and health system factors in shaping TB detection dynamics. Strengthening disaggregated surveillance and targeted case-finding strategies will be essential to ensure that post-pandemic recovery translates into more equitable TB control.
